# Objective Measurement of Walking Activity Using Wearable Technologies in People with Parkinson Disease: A Systematic Review

**DOI:** 10.3390/s22124551

**Published:** 2022-06-16

**Authors:** Mathias Baptiste Correno, Clint Hansen, Thomas Carlin, Nicolas Vuillerme

**Affiliations:** 1 Laboratory AGEIS, Université Grenoble Alpes, 38000 Grenoble, France; mathias.correno@gmail.com (M.B.C.); carlin.thom@gmail.com (T.C.); nicolas.vuillerme@univ-grenoble-alpes.fr (N.V.); 2 LabCom Telecom4Health, Orange Labs, Université Grenoble Alpes, CNRS, Inria, Grenoble INP-UGA, 38000 Grenoble, France; 3 Department of Neurology, Universitätsklinikum Schleswig-Holstein, 24105 Kiel, Germany; 4 Institut Universitaire de France, 75231 Paris, France

**Keywords:** Parkinson’s disease, walking activity, wearable devices, monitoring strategy, systematic review

## Abstract

Parkinson’s disease (PD) is a complex neurodegenerative disease with a multitude of disease variations including motor and non-motor symptoms. Quality of life and symptom management may be improved with physical activity. Due to technological advancement, development of small new wearable devices recently emerged and allowed objective measurement of walking activity in daily life. This review was specifically designed to synthesize literature on objective walking activity measurements using wearable devices of patients with PD. Inclusion criteria included patients with a diagnosis of PD and exclusion criteria included studies using animal models or mixed syndromes. Participants were not required to undergo any type of intervention and the studies must have reported at least one output that quantifies daily walking activity. Three databases were systematically searched with no limitation on publication date. Twenty-six studies were eligible and included in the systematic review. The most frequently used device was the ActiGraph GT3X which was used in 10 studies. Duration of monitoring presented a range from 8 h to one year. Nevertheless, 11 studies measured walking activity during a 7-day period. On-body sensor wearing location differed throughout the included studies showing eight positions, with the waist, ankle, and wrist being the most frequently used locations. The main procedures consisted of measurement of walking hours during a 2-day period or more, equipped with a triaxial accelerometer at the dominant hip or ankle. It is also important for further research to take care of different factors such as the population, their pathology, the period, and the environment.

## 1. Introduction

Neurodegenerative diseases such as Parkinson’s disease (PD) can lead to motor [1,2] and non-motor symptoms [3]. The latter also often occur in in the general elderly population but people with PD show a stronger decline in a number of cognitive domains when compared to age-matched healthy adults (e.g., executive, attentional, and visuospatial domains) [4]. Motor symptoms on the other hand express themselves as bradykinesia, rigidity, tremor, and eventually even affect the ability to walk or maintain balance [1,5]. The most common motor-related deficits are gait disorders [6,7] which can lead to a loss of independence and increase the incidence of falls [8]. In addition, people with PD suffer from impaired functional abilities [9,10,11], based on a reduced level of strength [11,12] and lower physical activity levels [13,14].

This systematic review focuses on the use of wearable technology as a method to monitor the relationship between walking in PD and between clinical rating scales (e.g., MDS-UPDRS III) [15]. A better understanding and representation of solutions for instrumented monitoring of walking activity in PD could help clinicians and researchers when designing interventions and trials. Consequently, this review aimed to identify and map available studies on the use of wearable technology for objectively measuring walking in people with PD.

## 2. Materials and Methods

This review complies with the PRISMA guidelines to provide an evidence-based minimum set of items for reporting in systematic reviews [16]. The use of wearable technology to detect walking activity is explored to map the state of evidence and to identify potential research gaps [17]. The protocol of this current review has been registered in the PROSPERO (CRD42020210866) prospective register of systematic reviews and published in July 2021 [18].

Detailed information about the eligibility criteria, population, measurement tools, experimental procedures, measured outcomes, data sources and search strategy, study selection, data extraction, and data synthesis can be found in [18]. Briefly, peer-reviewed scientific original articles on patients with PD were included in this review. Participants were not required to undergo any type of intervention and the wearable technology could contain any combination of electronic or spring-levered uni- or multiaxial accelerometer, gyroscope, magnetometer, or barometer. The experimental protocol could take place in a laboratory or in a free-living environment. The reported outcomes contained parameters quantifying daily walking activity (e.g., daily step count or distance travelled). After completion of the screening process, two reviewers (M.C. and C.H.) independently extracted the data from each included article including wearing location, technology used, and the methodology to capture daily walking activity.

## 3. Results

### 3.1. Study Selection

**Figure 1:**
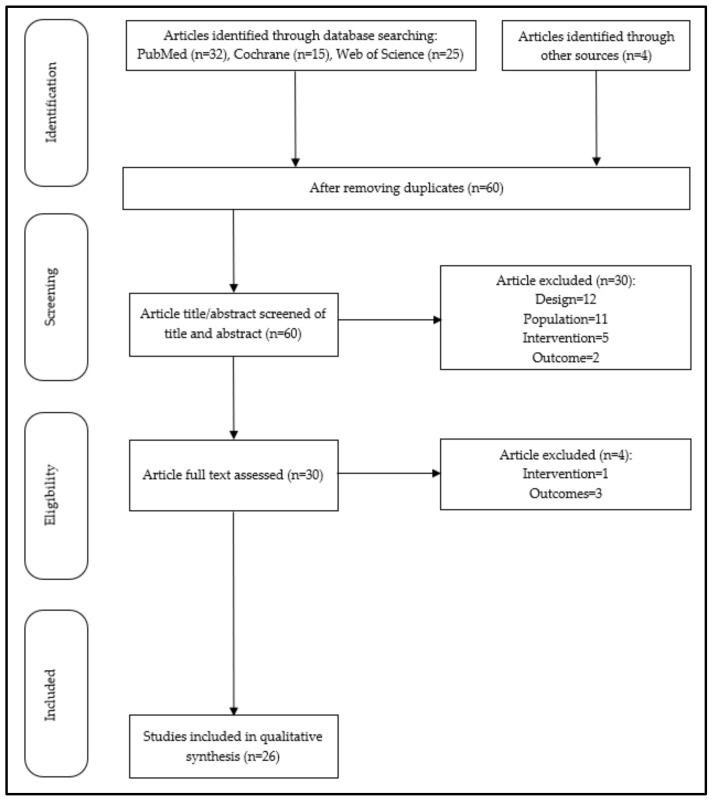
Flowchart of study selection. A total of 26 studies were included according to the eligible criteria in the synthesis.

A total of 72 studies were identified. Additional content (*n* = 4) was included with this selection from other sources and the article selection process is detailed in a flowchart (Figure 1). After removing duplicates, a total of 60 studies were screened based on the abstracts. This first step excluded 50% of abstracts according to inclusion and exclusion criteria. Then, the thirty eligible articles remaining were assessed on full text. Four studies were excluded after full reading and finally twenty-six studies were eligible and included in the systematic review.

### 3.2. Study Characteristics

**Figure 2:**
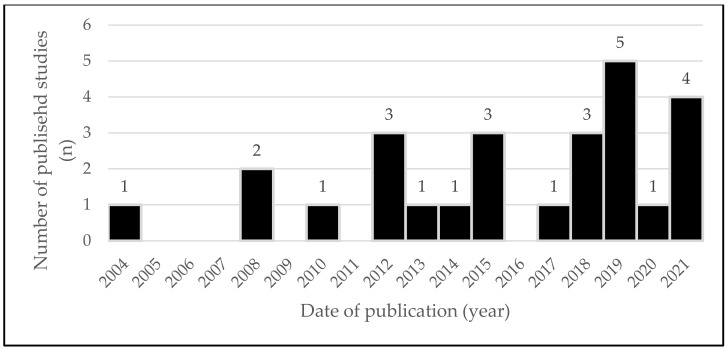
Number of published studies per year (*n*).

**Figure 3:**
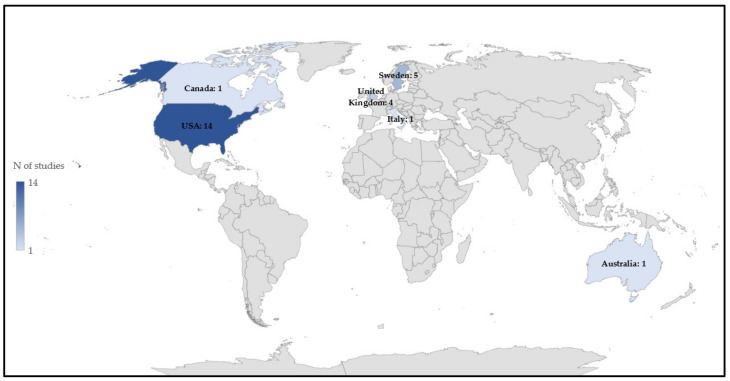
Number of eligible papers published per country (*n*).

The earliest research dates back to 2004, and interest has sparked over the last years with no less than 14 studies published since 2017 (Figure 2). Fifty percent of the included studies originates from the USA followed by Sweden and the United Kingdom (Figure 3).

The objective of the 26 studies ranged from observational (*n* = 18, 69.2%) [19,20,21,22,23,24,25,26,27,28,29,30,31,32,33,34,35,36] to interventional studies (*n* = 6, 23.1%) [37,38,39,40,41,42] including pre- and post-measurements or the comparison of walking activity parameters with a control group. Two studies can be considered as proof of concept [28,43]. Six main objectives have been pursued in the included studies:

1. To evaluate ambulatory activity [21,23,26,29,30,31,33,35,36,37];
2. To assess the effect of a training program [27,38,39,41,42,44];
3. To examine the relationship between two variables [20,22,24,25,28,32];
4. To compare accuracy of wearable sensors according to on-body location [40] and environment [34];
5. To compare PD parameters assessed with the ActiGraph GT3X+ (AGT3X) accelerometer and processed with two different filter settings [40];
6. To investigate the reliability and validity of a device [43].

### 3.3. Sample Characteristics

The 26 studies combined a sample size of 1263 people with PD and healthy controls (*n* = 317). Table A1 in Appendix A, reporting participant’s characteristics, shows mean age ranging from 54.9 [30] to 73.4 [23] years old, mean height (160 [29]–172 cm [19]), a mean weight (69.2 [36]–77 kg [19]), and a mean BMI (24.2 [21]–28.07 [42]) among studies reporting these anthropometric data. Among all participants, 584 females and 679 males were included in this review. Disease severity was assessed using Hoehn and Yahr (*n* = 20, 76.9%) [19,20,22,23,24,26,27,28,29,30,31,33,34,35,36,37,38,41,42,44] and MDS-UPDRS Part III (*n* = 18, 69.2%) [19,20,22,24,25,26,27,29,31,32,34,35,36,37,38,39,41,42]. If reported, disease duration ranged from 0.8 [37] to 12.5 years [30] (Table A2).

### 3.4. Type of Sensors

The most frequently used wearable device was the AGT3X [19,20,21,22,23,36,37,38,39,40] used in 10 studies (38.5%). StepWatch Activity Monitors (SAM) were used in seven studies (26.9%): the StepWatch (*n* = 3, 11.5%) [24,25,43], the StepWatch 3 (*n* = 3, 11.5%) [26,27,41], and the StepWatch 4 (*n* = 1, 3.8%) [28]. ActivPAL (*n* = 1, 3.8%) [29,30] and FitBit Charge HR (n = 1, 3.8%) [31,32] were both used in two studies. Finally, the following wearable devices were employed only in one study each: ActiTrainer (*n* = 1, 3.8%) [33], Axivity AX3 (*n* = 1, 3.8%) [42], BioStampRC (*n* = 1, 3.8%) [34], FitBit Zip (*n* = 1, 3.8%) [44], Garmin Forerunner 405 GPS Watch (*n* = 1, 3.8%) [33], and PAMSys (*n* = 1, 3.8%) [35]. Table A3 gives a summary of study characteristics and provides information on device, wearing location, and length of monitoring.

**Figure 4:**
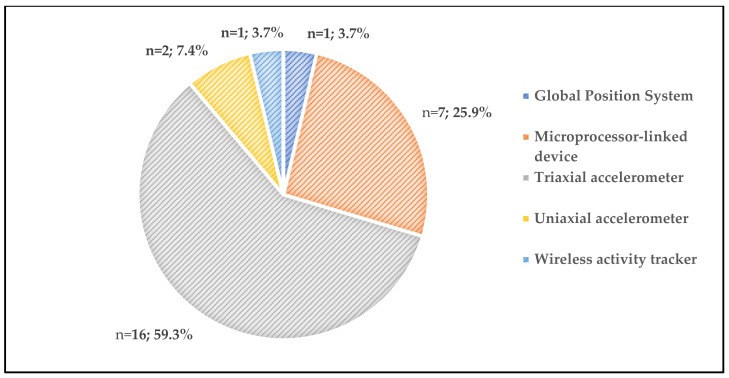
Proportion type of wearable devices used in the included studies (*n*; %).

Figure 4 illustrates proportion of sensors. The most commonly used sensors were triaxial accelerometers (*n* = 16, 61.5%) [19,20,21,22,23,31,32,33,34,35,36,37,38,39,40,42], followed by microprocessor linked devices which were used in seven studies (26.9%) [24,25,26,27,28,41,43]. Furthermore, two (7.7%) studies conducted assessments with uniaxial accelerometers [29,30]. Two studies assessed daily physical activity with a wireless activity tracker (*n* = 1, 3.8%) [44] and a Global Position System (*n* = 1, 3.8%) [33].

### 3.5. Outcomes of Interest

In this review, 24 studies linked to the use of wearable devices in the context of walking activity were included. According to inclusion criteria, all studies somehow measured walking activity. The included studies have reported various outcomes such as steps per day, gait speed, and time spent walking but also more general outcomes such as sedentary time, active time, activity counts, walking activity, light physical activity, moderate to vigorous physical activity, and high intensity physical activity.

**Figure A1:**
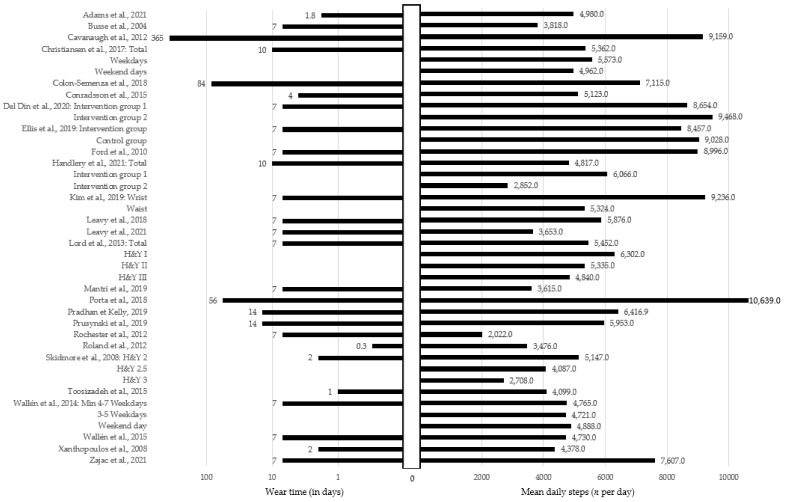
Wear time (in days) and mean daily step (*n* per day) of included studies.

Outcomes of interest are presented in Table A4. Participants with PD had a large range of 2022 to 10,639 steps/day (Figure A1). Eight studies evaluated gait speed of the population [19,26,31,34,35,36,38,43], including self-selected speed and maximal speed [26,31].

**Figure 5:**
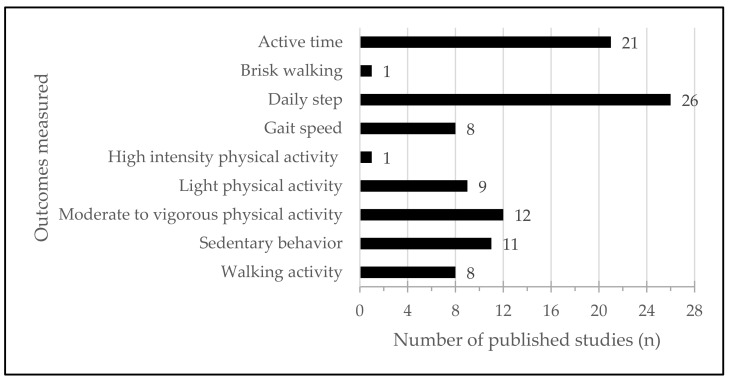
Outcomes measured in included studies (*n*).

Figure 5 allows a better understanding of the outcomes measured. Sedentary behavior was explored in 11 studies (42.3%) [19,21,23,26,31,32,33,34,35,39,40], and measured in min·day^−1^ or in min·hours^−1^ of wear time. Finally, active time is an outcome of interest due to the important number of articles which evaluated active time (*n* = 21, 80.8%) [19,20,21,22,23,24,26,27,29,30,31,32,33,34,35,36,37,39,40,42,44]. Active time was identified by: active time, activity counts, walking activity, light physical activity, moderate to vigorous physical activity, high intensity physical activity, or brisk walking.

### 3.6. Monitoring Protocol

**Figure 6:**
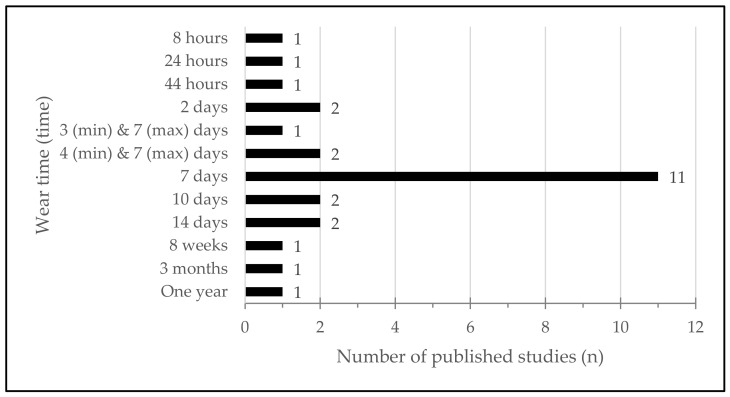
Wear time protocol of included studies.

Duration of monitoring (Figure 6) was very incongruent and presented a range from eight hours [33] to one year [26]. However, 11 studies (42.2%) measured walking activity for a 7-day period [19,21,22,27,28,29,30,40,41,42,43].

**Figure 7:**
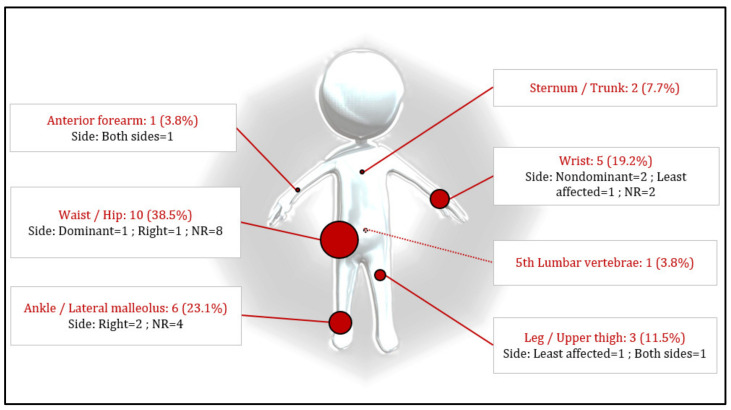
Location and percentage of devices according to method description.

The device location changed within the included studies, resulting in eight different on-body locations. The device on-body location is shown in Figure 7, highlighting three main device locations: waist (*n* = 10, 38.5%) [19,21,22,23,33,37,38,39,40,44], ankle (*n* = 6, 23.1%) [29,30,43,45,46,47], and wrist (*n* = 5, 19.2%) [19,31,32,33,36]. Five studies (19.2%) [32,33,34,36,41] did not provide details about the laterality of the device location.

In the panel of included studies, 12 recorded physical activity also during the night [19,23,27,30,31,32,34,35,36,40,42,43]. Other studies kept their focus only on daytime hours [20,21,22,24,25,26,28,33,37,38,39,41]. Two studies did not provide detailed information about the wearing time [29,44].

Only one study [35] mentioned declared specifically to have recorded weekdays, while four studies [24,25,33,34] did not specify whether weekdays or the weekend was measured. Only two studies indicated clearly if they measured weekdays or weekend days and presented results for each period [23,37].

Three studies compared baseline to final measures [26,30,38]. Five studies evaluated PD patients and healthy controls [31,32,33,34,43], while PD patients were also compared to other groups: ActiGraph wearers vs. non-ActiGraph wearers [22], mHealth vs. active control [41], and older fallers vs. fallers with mild cognitive impairment [42]. Nine studies (34.6%) reported the measured outcomes without additional comparison to other groups, conditions, or outcomes [21,23,24,25,27,28,37,40,44].

## 4. Discussion

The aim of this systematic literature review was to document the use of wearable technology for objective measurement of walking activity in people with PD [18].

Based on the included studies, triaxial accelerometers were the most represented wearable devices [19,20,21,22,23,31,32,33,34,35,36,37,38,39,40,42], followed by microprocessor-linked devices [24,25,26,27,28,41,43]. Both devices allow for an investigation of walking as well as physical activity measures depending on the underlying software/algorithm packages. At this point it has to be mentioned that some of the stated devices only provide raw sensor data, i.e., accelerations that need custom post-processing to compute the desired parameters. Systems such as the AGT3X sensor or the FitBit devices are extensively used to measure step numbers and physical activity intensity even though the parameters are not necessarily validated in the studied cohort. As an example, Riel et al. (2016) validated the ActiGraph step count for post-stroke survivors at specific walking velocities [45], but not yet for patients with PD [46]. Nonetheless, the devices prove beneficial when estimating steps under free-living conditions [47], similarly to the FitBit at least in healthy female adults [48]. Overall, commercial wearable devices are accurate within their application specification [49] and it is necessary to understand for which clinical cohort the devices are actually validated and if they can be used in the clinical setting. Furthermore, the implementation of wearables in clinical settings requires formation and training of health professionals and patients when using wearable activity trackers [50,51].

While some wearable devices may be of preference for research, Bodine and Gemperle (2003) highlighted that the function of any wearable tool must outweigh any physical or social discomfort felt when wearing it [52]. This directly influences the sensor placement, which as a consequence affects the reliability and validity of the sensor outcomes. Kim et al. (2019) compared the number of steps recorded by an AGT3X attached at the wrist (least affected hand) and one attached at the waist (right hip) in a cohort of PD patients. At moderate speed (1.05–1.3 m/s), results showed an overestimation of daily step count for the wrist and an underestimation for the device recording at waist level. Similarly, in the laboratory environment, waist-worn sensors showed higher accuracy compared to wrist worn sensors, but all activity monitors underestimated the number of steps [53].

Yet, most of our walking activity happens outside the laboratory and the included studies provide a range from eight hours [33] to one year [26] for the monitoring period. While none of the studies justified their monitoring period, Cavanaugh et al. (2012) provided evidence that walking activity of PD patients did not differ if you measure longitudinal data over the course of one year, compared to shorter monitoring periods. Their findings are supported by Paul et al. (2016) who determined that two consecutive days of monitoring are sufficient to estimate daily activity reliably during a representative week in people with PD [54]. Moreover, the amount of ambulatory activity was greater on weekdays than weekends in this study which is supported by Christiansen et al. (2017) who found a significant difference for number of steps between weekdays and weekend days. This does not only hold true for PD patients but also for adolescent girls who present a greater activity and greater moderate vigorous intensities on weekdays than on weekends [55]. The concept of weekend warriors and couch potatoes is well established and depending on the employment, educational level, and household income, activity behaviors and patterns during the weekend differ, which is supported by [56,57].

However, in PD patients, the situation is more complicated. While overweight/obesity are common [58], age, gender, education, disease duration, Hoehn and Yahr stage, UPDRS-II and UPDRS-III scores, and dosage of levodopa do not correlate with physical activity [59].

In addition, behavioral and environmental factors are known to affect physical performance. A relationship between sleep and physical activity exists, suggesting that sleep quality could deteriorate walking activity [60]. Dog owners also showed greater walking activity compared to people not owning a dog [61,62]. However, walking activity decreased significantly with increasing wind speed, precipitation, and humidity [63].

To conclude, the selection of an appropriate sensor ultimately depends on the purpose of the study, methodological considerations, and the population characteristics [64].

Even though this systematic review highlights major findings to evaluate walking activity, these findings must be interpreted with caution. Only the outcome steps per day was consistent throughout studies, and all other reported outcomes were exclusively reported by the respective study. Future research could analyze these individual outcomes to improve our understanding of the role of the device when measuring walking activity. Data processing techniques vary greatly and make the comparison between studies rather difficult. In addition, this review already includes a large sample of PD patients; however, the variability of PD severity between each group increases the difficulty to compare the specified protocols.

## 5. Conclusions

To conclude, this systematic review documents the most frequently used wearable devices as well as data collection procedures and data processing in PD patients. Walking activity is mainly assessed during a 2-day period or more, using a triaxial accelerometer preferably located at the hip or ankle. These findings may be taken into account when evaluating walking activity in PD patients.

## Data Availability

All data (articles) are available upon reasonable request to the corresponding author C.H.

## Appendix A

**Table A1:** Characteristics of patients and anthropometric information.

Study	Sample Size	Age, Years (SD)	Gender (n Male, %)	Height, cm (SD)	Weight, kg (SD)	BMI, kg/m^2^ (SD)
**Busse et al., 2004** [43]	10	67.1 (8.2)	7 (70.0%)			
**Skidmore et al., 2008** [24]	24 (26)	70.0 (9.0)	18 (69.2%)			
**Xanthopoulos et al., 2008** [25]	16	71 (11)	10 (62.5%)			
**Ford et al., 2010** [27]	12	67.2	11 (91.7%)			
**Cavanaugh et al., 2012** [26]	33	67.06 (8.75)	22 (66.7%)			
**Rochester et al., 2012** [30]	14	54.9 (9.5)	9 (52.9%)			
**Roland et al., 2012** [33]	Total = 15 Non-Frail = 5 Pre-Frail = 5 Frail = 5	Total = 65.0 (9.0) NF = 69 (1) PF = 65 (10) F = 63 (11)	0 (0.0%)			NF: 26.31 (5.6) PF: 22.95 (4.3) F: 25.06 (4.3)
**Lord et al., 2013** [29]	89	67.3 (9.9)	62 (69.7%)	160 (7)		
**Wallen et al., 2014** [40]	66	73.1 (5.8)	38 (57.6%)	171.0 (8.8)	74.8 (13.8)	25.5 (3.8)
**Conradsson et al., 2015** [38]	47	72.9 (6.0)	28 (59.8%)	171.8 (9.2)	75.8 (14.5)	
**Toosizadeh et al., 2015** [35]	15	71.2 (6.3)	8 (53.0%)	164.3 (10.9)	74.9 (15.3)	27.5 (6.5)
**Wallen et al., 2015** [23]	95	73.4 (5.7)	53 (55.8%)	171.4 (9.3)	76.6 (14.2)	25.8 (3.7)
**Christiansen et al., 2017** [37]	113	64.3 (8.6)	63 (55.8%)			26.9 (4.2)
**Colón-Semenza et al., 2018** [44]	Total = 10 Peer Coach = 5 Peer Mentee = 5	PC = 64.6 (4.04) PM = 63.4 (2.06)	PC = 3 (60%) PM = 3 (60%)			
**Leavy et al., 2018** [20]	49	75.0 (5.9)	21 (49.1%)			25.7 (3.5)
**Porta et al., 2018** [36]	18	68.0 (10.8)	8 (44.4%)	165.6 (7.9)	69.2 (9.4)	
**Ellis et al., 2019** [41]	Total = 51	Total = 64.1 (9.5)	Total = 28 (74.5%)			
	mHealth = 26	mH = 64.8 (8.5)	mH = 15 (57.7)			
	Active Control = 25	AC = 63.3 (10.6)	AC = 13 (52.0)			
**Kim et al., 2019** [19]	46	68.0 (7.9)	32 (69.6%)	172 (9.4)	77 (15)	
**Mantri et al., 2019** [22]	Total = 63 ActiGraph Wearer = 30 Non-ActiGraph Wearer = 33	Total = 70 AW = 70 NAW = 70	Total = 3 (95.2%) AW = 2 (93.3%) NAW = 1 (97.8%)			AW = 26.9 NAW = 26.5
**Pradhan and Kelly, 2019** [31]	30	68.6	11 (36.7%)			
**Prusynski et al., 2019** [32]	25	69.0 (6.0)				
**Del Din et al., 2020** [42]	128	71.68 (6.43)	81 (83.0%)			28.07 (3.62)
**Adams et al., 2021** [34]	17	66.4 (11.3)	10 (58.8%)			
**Handlery et al., 2021** [39]	Total = 110	Total = 65	Total = 62 (56.4%)			
	S+ = 74	S+ = 61.5	S+ = 45 (60.8%)			
	S− = 36	S− = 70.5	S− = 17 (47.2%)			
**Leavy et al., 2021** [21]	89	71.0 (6.0)	48 (54.0%)			24.2 (3.5)
**Zajac et al., 2021** [28]	69	67.5 (8.7)	40 (58.0%)			

PC = peer coach. PM = peer mentee. mH = mobile health. AC = active control. S+ = more than 4200 steps/day. S− = less than 4220 steps/day. AW = ActiGraph wearer. NAW = non-ActiGraph wearer. NF = non-frail. PF = pre-frail. F = frail.

**Table A2:** Characteristics of patients concerning cognitive state.

Study	Duration Disease, Years (SD)	MDS-UPDRS III: Motor Examination Score (SD)	Hoehn and Yahr (SD)	Medications (LEDD in mg, SD)
**Busse et al., 2004** [43]				
**Skidmore et al., 2008** [24]	7.5 (3.8)	On: 35 (10); Off: 28 (10)	2 = 9	
			2.5 = 6	
			3/4 = 9	
**Xanthopoulos et al., 2008** [25]	7.0 (4.2)	29 (11)	NA	
**Ford et al., 2010** [27]		12.4	1 = 4	
			1.5 = 1	
			2 = 4	
			2.5 = 1	
			3 = 2	
**Cavanaugh et al., 2012** [26]	4.44 (4.21)	Baseline: 28.18 (8.56)	Baseline: 2 = (1–3)	Baseline = 303.03 (294.38)
		One year: 28.52 (11.71)	One year: 2 = (1.5–3)	One year = 423.49 (359.66)
**Rochester et al., 2012** [30]	12.5 (6.4)		3 = 13	Baseline = 1387.35 (415.9)
			4 = 4	6 months follow-up = 1056.8 (293.0)
**Roland et al., 2012** [33]			NF = 1.83 (0.8)	Yes
			PF = 1.86 (0.6)	
			F = 2.50 (0.4)	
**Lord et al., 2013** [29]		25 (10.7)	1 = 20 (22.5 %)	174.6 (124.1)
			2 = 51 (57.3 %)	
			3 = 18 (20.2 %)	
**Wallen et al., 2014** [40]			NR	
**Conradsson et al., 2015** [38]	6.0 (5.1)	36 (10)	2 = 20 (43%)	581 (295)
			3 = 27 (57%)	
**Toosizadeh et al., 2015** [35]	5.9 (5.3)	34.8 (13.9)	2.9 (0.9)	517 (380)
**Wallen et al., 2015** [23]	5.9 (5.0)		2 = 41	
			3 = 54	
**Christiansen et al., 2017** [37]	0.8 (0.9)	21.1 (8.8)	1 = 29 (25.6%)	No medication
			2 = 84 (74.3%)	
**Colón-Semenza et al., 2018** [44]	PC: 5.2 (1.24) PM: 6.2 (2.2)		1: PC = 3/PM = 1	
			2: PC = 1/PM = 3	
			3: PC = 1/PM = 1	
**Leavy et al., 2018** [20]	6	40 (10.9)	2 = 22 (45%)	635 (306)
			3 = 27 (55%)	
**Porta et al., 2018** [36]	9.9 (6.0)	17.8 (9.6)	1.9 (0.4)	Levodopa: MAO-B inhibitors (*n* = 11), Rasagiline (*n* = 8), Safinamide (*n* = 3)
**Ellis et al., 2019** [41]	Total: 4.8 (3.1) mH: 5.9 (3.5) AC: 3.7 (2.1)	Tot = 29.6 (10.0) mH = 31.6 (10.7) AC = 27.6 (9.1)	1: Tot = 1 (2%); mH = 1 (3.9); AC = 0 (0)	
			1.5: Tot = 2 (3.9%); mH = 1 (3.9); AC = 1 (4.0)	
			2: 38 (74.5%); mH = 20 (76.9); AC = 18 (72.0)	
			2.5: Tot = 7 (13.7%); mH = 4 (15.4); AC = 3 (12.0)	
			3: Tot = 3 (5.9%); mH = 0 (0); AC = 3 (12.0).	
**Kim et al., 2019** [19]	7.6 (6.8)	34.4 (13.2)	1 = 4 (8.7%)	Yes
			2 = 33 (71.7%)	
			3 = 7 (15.2%)	
			4 = 2 (4.3%)	
**Mantri et al., 2019** [22]	AW: 4	AW = 16.5	AW = 2	
	NAW: 3	NAW = 21	NAW = 2	
**Pradhan and Kelly, 2019** [31]	7.8 (5.0)	12.9 (10.3)	1 = 18	
			2 = 12	
**Prusynski et al., 2019** [32]		12 (9)		
**Del Din et al., 2020** [42]		30.37 (16.96)	2 = 48%	
			2.5 = 10%	
			3 = 42%	
**Adams et al., 2021** [34]	4.8 (4.0)	20.9 (7.9)	1.9 (0.8)	
**Handlery et al., 2021** [39]	Total: 0	Tot = 19		Yes
	S+: 0	S+ = 18.5		
	S−: 0	S− = 22		
**Leavy et al., 2021** [21]	6.0 (4.3)			580 (291)
**Zajac et al., 2021** [28]			2 = 27 2.5 = 30 3 = 12	

PC = peer coach. PM = peer mentee. mH = mobile health. AC = active control. S+ = more than 4200 steps/day. S− = less than 4220 steps/day. AW = ActiGraph wearer. NAW = non-ActiGraph wearer. NF = non-frail. PF = pre-frail. F = frail.

**Table A3:** Characteristics of sensors and conditions of data acquisition.

Study	Name of Sensor	Manufacturer	Type of Sensor	N of Sensor	Wearing Location	Side	Duration	Days Included	Wear Time
**Busse et al., 2004** [43]	SAM	Cymatech, Seattle, WA, USA	Microprocessor-linked device	1	Right lower limb above the lateral malleolus	NR	7 days × 2	WD-WED	Continuously. Except during water-related activities.
**Skidmore et al., 2008**	SAM	Cyma Corporation, Mountlake Terrace, WA, USA	Microprocessor-linked device	1	Ankle	NR	2 days	NR (WD)	Continuously. Except during water-related activities and sleep.
**Xanthopoulos et al., 2008** [25]	SAM	Cyma Corporation, Mountlake Terrace, WA, USA	Microprocessor-linked device	1	Over the right lateral malleolus	NR	2 days	NR (WD)	Continuously. Except during water-related activities and sleep.
**Ford et al., 2010** [27]	SAM 3	Orthocare Innovations, Mountlake Terrace, WA, USA	Microprocessor-linked device	1	Ankle	NR	7 days	WD-WED	Continuously. All periods.
**Cavanaugh et al., 2012** [26]	SAM 3	Orthocare Innovations, Mountlake Terrace, WA, USA	Microprocessor-linked device	1	Ankle	NR	1 year	WD-WED	Continuously. During customary activity, including exercise, waking hours. Except during water-related activities.
**Rochester et al., 2012** [30]	ActivPAL	PAL Technologies, Glasgow, Scotland	Uniaxial accelerometer	1	NR	NR	7 days	WD-WED	Continuously. Except during water-related activities.
**Roland et al., 2012** [33]	(1) ActiTrainer (2) Garmin Forerunner 405 GPS watch	(1) ActiGraph, LLC, Fort Walton Beach, FL, USA (2) Garmin International Inc., Olathe, KS, USA	(1) Triaxial accelerometer (2) GPS	2	(1) Waist (2) Wrist	(1) Dominant (2) NR	8 h	NR (WD)	Setup of daily physical activity monitors (accelerometer, GPS) were completed at the participant’s home in the morning (8–10 a.m.). All PD participants were assessed between 1 and 2 h post anti-Parkinson’s medication. The accelerometer, GPS, and physical activity logbook were collected approximately 7 h later (between 4–7 p.m.).
**Lord et al., 2013** [29]	ActivPAL	PAL Technologies, Glasgow, Scotland	Uniaxial accelerometer	1	On the upper thigh	NR	7 days	WD-WED	NR
**Wallen et al., 2014** [40]	GT3X+	ActiGraph, Pensacola, FL, USA	Triaxial accelerometer	1	Waist (at hip level above the anterior superior iliac spine)	NR	3 days (min) and 7days d (max)	WD-WED	Continuously. Except during water-related activities and sleep.
**Conradsson et al., 2015** [38]	GT3X+	ActiGraph, Pensacola, FL, USA	Triaxial accelerometer	1	Waist	NR	4 days (or more)	WD-WED	At least 9 h/day.
**Toosizadeh et al., 2015** [35]	PAMSys	PAMSys, BioSensics, Boston, MA, USA	Triaxial accelerometer	1	Sternum	NA	1 day	WD	Continuously.
**Wallen et al., 2015** [23]	GT3X+	ActiGraph, Pensacola, FL, USA	Triaxial accelerometer	1	Waist (at hip level)	NR	7 days	WD-WED	Continuously. Except during water-related activities and sleeping.
**Christiansen et al., 2017** [37]	GT3X+	ActiGraph, Pensacola, FL, USA	Triaxial accelerometer	1	Waist	NR	10 days	WD-WED	10 h of valid wear time/day minimum, 90 min of non-wear/day maximum. At least 3 weekdays and 1 weekend day of valid wear time.
**Colón-Semenza et al., 2018** [44]	FitBit Zip	Fitbit Inc., San Francisco, CA, USA	Wireless activity tracker	1	Waist	NR	8 weeks	WD-WED	NR
**Leavy et al., 2018** [20]	GT3X+	ActiGraph, Pensacola, FL, USA	Triaxial accelerometer	1	NR	NR	4 days (min) and 7 days (max)	WD-WED	<540 min/day.
**Porta et al., 2018** [36]	GT3X+	ActiGraph, Pensacola, FL, USA	Triaxial accelerometer	1	Wrist	Nondominant	3 months	WD-WED	Continuously. 24 h/24 h. Except during water-related activities.
**Ellis et al., 2019** [41]	SAM 3	Orthocare Innovations, Mountlake Terrace, WA, USA	Microprocessor-linked device	1	Leg	Least severe impairment	7 days × 4	WD-WED	Continuously. During waking hours. Except during water-related activities.
**Kim et al., 2019** [19]	GT3X+	ActiGraph, Pensacola, FL, USA	Triaxial accelerometer	2	(1) Wrist (2) Waist	(1) Least affected hand (2) Right hip	7 days	WD-WED	Continuously. 24 h/24 h.
**Mantri et al., 2019** [22]	GT3X+	ActiGraph, Pensacola, FL, USA	Triaxial accelerometer	1	Waist	NR	7 days	WD-WED	During walking hours.
**Pradhan and Kelly, 2019** [31]	Fitbit Charge HR	Fitbit Inc., San Francisco, CA, USA	Triaxial accelerometer	1	Wrist	NR	14 days	WD-WED	Continuously. Except for the time needed to charge the device and during water-related activities.
**Prusynski et al., 2019** [32]	Fitbit Charge HR	Fitbit Inc., San Francisco, CA, USA	Triaxial accelerometer	1	Wrist	Nondominant	14 days	WD-WED	Continuously. Except during water-related activities and for the time needed to charge the device.
**Del Din et al., 2020** [42]	Axivity AX3	Axivity AX3, York, UK	Triaxial accelerometer	1	Fifth lumbar vertebra	NA	7 days	WD-WED	Continuously. Participants were asked to continue their daily activities as usual and not to change their routine.
**Adams et al., 2021** [34]	BioStampRC	MC10 Inc., Cambridge, MA, USA	Triaxial accelerometer	5	(1) One on each anterior thigh. (2) One on each anterior forearm. (3) One on the trunk	Both sides	44 h	NR (WD)	Continuously.
**Handlery et al., 2021** [39]	GT3X+	ActiGraph, Pensacola, FL, USA	Triaxial accelerometer	1	Waist	NR	10 days × 6	WD-WED	During waking hours.
**Leavy et al., 2021** [21]	GT3X+	ActiGraph, Pensacola, FL, USA	Triaxial accelerometer	1	Hip	NR	7 days	WD-WED	Continuously. 4 valid days min. 9 h or more of wear time/day.
**Zajac et al., 2021** [28]	SAM 4	Orthocare Innovations, Mountlake Terrace, WA, USA	Microprocessor-linked device	1	Above the lateral malleolus	NR	7 days	WD-WED	Continuously. During all waking hours. Except during water-related activities.

NR = not reported. WD = weekdays. WED = weekend days. SAM = StepWatch Activity Monitor.

**Table A4:** Main outcomes of the included studies.

Study	Comparison	Steps/Day (SD)		Gait Speed (m/s)	Sedentary Time (SD)	Active Time
		Baseline (If Possible)	End			
**Busse et al., 2004** [43]	HC	/	3818	0.99 (0.16)		
**Skidmore et al., 2008** [24]	No comparison	/	Stage 2 = 5147 (1903) Stage 2.5 = 4087 (1286) Stages 3/4 = 2708 (1155)			**Walking activity (steps/h):** HY 2 = 20.7 (9.0)/HY 2.5 = 12.9 (3.9)/HY 3–4 = 9.6 (4.5)
**Xanthopoulos et al., 2008** [25]	No comparison	/	4378 (2057)			
**Ford et al., 2010** [27]	No comparison	/	8996 (3466)			**Walking activity (min/d):** 322 (88)
**Cavanaugh et al., 2012** [26]	Baseline vs. one year	10,261.15 (4332.56)	9159.44 (3534.21)	**Max speed:** Baseline = 1.77 (0.50) End = 1.74 (0.53)	**Sedentary time (%):** Baseline = 78.53 (6.93) End = 80.14 (5.90)	**MVPA (min/day):** Baseline = 22.49 (24.16)/End = 16.07 (18.68)
**Rochester et al., 2012** [30]	Baseline vs. 6 months follow-up	2258.50 (1373)	2022.40 (1147.20)			**Activity count (n):** T1 = 315.3 (42.9)/T2 = 229.5 (47.4)
**Roland et al., 2012** [33]	HC	/	3476 (2814)		**Sedentary time (%):** 61.70 (14.1)	**Active time (%):** 32.20 (10.6) **MLTA questionnaire:** 3052.3 (1611.6)
**Lord et al., 2013** [29]	Control group	/	Total = 5452 (2501) HYI = 6302 (6302) HYII = 5335 (2716) HYIII = 4840 (1851)			**Total time spent walking (%):** Total = 5.1 (2)/HYI = 5.8 (1.8)/HYII = 5.1 (2.2)/HYIII = 4.4 (1.6)
**Wallen et al., 2014** [40]	No comparison	/	4730 (3210)		**Sedentary time (min/d):** 612 (103)	**LPA I (min/d):** 138 (64) **LPA II (min/d):** 31 (24) **MVPA (min/d):** 17 (22)
**Conradsson et al., 2015** [38]	Baseline Control group	4842 (528)	5123 (545)	Baseline: 1.19 (0.03) End: 1.28 (0.03)		
**Toosizadeh et al., 2015** [35]	Control group	/	4099 (2673)	0.66 (0.11)	**Sitting (%):** 44.11 (16.39) **Standing (%):** 14.40 (7.79) **Lying (%):** 35.36 22.01)	**Walking activity (%):** 6.02 (3.83) **Walking episodes (n):** 381 (205) **Max steps (n):** 189 (290)
**Wallen et al., 2015** [23]	No comparison	/	Minimum of 4/7 d/week = 4765 3–5 Weekdays = 4721 Weekend days = 4888		**Sedentary time (min/d):** M4/7 = 588.9 3–5 WD = 593.9 WE = 584.5	**LPA (min/d):** M4/7 = 140.6/3–5 WD = 143.0/WE = 142 **MPALS (min/d):** M4/7 = 30.1/3–5 WD = 30.7/WE = 31.3 **MVPA (min/d):** M4/7 = 16.4/3–5 WD = 15.2/WE = 18.4 **Activity count (n):** 135,721 (92 368)
**Christiansen et al., 2017** [37]	No comparison	/	Total = 5362 (2890) WD = 5573 (3144) WE = 4692 (2800)			**Activity count (n):** Total = 345,870 (141,796)/WD = 353,196 (154,472)/WE = 321,456 (141,173)
**Colón-Semenza et al., 2018** [44]	No comparison	5428 (2440)	7115 (1291)			**Activity (min/week):** Baseline = 199 (95)/End = 282 (83)
**Leavy et al., 2018** [20]	Control group	/	3653			**LPA (min/d):** 197 (72) **MVPA (min/d):** 47 (34)
**Porta et al., 2018** [36]	Other studies	/	Total = 10,639 06::00–12:00 = 4313 12:00–18:00 = 3437 18:00–22:00 = 2889	Baseline = 1.18 End = 1.18		**MVPA (%):** 06:00–12:00 = 43.2/12:00–18:00 = 36.3/18:00–22:00 = 31.4
**Ellis et al., 2019** [41]	mHealth vs. active control	mH = 8478 (3699) AC = 8902 (2967)	mH = 8457 (3184) AC = 9028 (3366)			
**Kim et al., 2019** [19]	Wrist vs. waist	/	Wrist = 9236 (3812) Waist = 5324 (2800)	**Time spent walking < 1.04 (%):** Wrist = 71 Waist = 95 **Time spent walking 1.05–1.30 (%):** Wrist = 29 Waist = 4 **Time spent walking > 1.31 (%):** Wrist = 0 Waist = 1	**Sedentary time (%):** Wrist = 38 (13) Waist = 70 (11)	**LPA (%):** Wrist = 51 (9)/Waist = 28 (10) **MVPA (%):** Wrist = 11 (8)/Waist = 2 (2) **Activity count (n):** Wrist = 872 590 (349 148)/Waist = 186 491 (101 989)
**Mantri et al., 2019** [22]	ActiGraph Wearers vs. Non-ActiGraph Wearers	/	3615			**MVPA (min):** 8.1 (Q = 2.2–23.2)
**Pradhan and Kelly, 2019** [31]	HC	/	6416.9 (2795.5)	**Self-selected speed:** 1.5 (0.3) **Max speed:** 2.2 (0.4)	**Sedentary time (min/d)/(%):** 803.7 (154.9)/78.9 (7.8)	**LPA (%):** 17.8 (6.6) **MVPA (%):** 2.7 (1.9)
**Prusynski et al., 2019** [32]	HC	/	5953 (2363)		**Sedentary time (min/d):** 846 (122)	**LPA (min):** 172 (60) **MVPA (min):** 15 (11) **HIPA (min):** 19 (14)
**Del Din et al., 2020** [42]	Older fallers vs. fallers with MCI	I1 = 8874 (4538) I2 = 9000 (4535)	I1 = 8654 (4638) I2 = 9468 (5576)			**Total Walking Time per Day (min):** Baseline: I1 = 117.3 (59.1)/I2 = 117.8 (62.3) End: I1 = 116.2 (65.1)/I2 = 127.9 (84.3) **Percentage of Walking Time (%):** Baseline: I1 = 8.2 (4.1)/I2 = 8.2 (4.3) End: I1 = 8.1 (4.5)/I2 = 8.9 (5.9) **Activity count (act/d):** Baseline: I1 = 233 (106)/I2 = 242 (110) End: I1 = 229 (116)/I2 = 254 (107)
**Adams et al., 2021** [34]	HC	/	4980	0.91	**Lying (h/d):** 9.1 **Sitting (h/d):** 10.7 **Standing (h/d):** 3.3	**Walking activity (h/d):** 0.9 (0.5–1.3)
**Handlery et al., 2021** [39]	>4200 steps/day (S+) vs. <4200 steps/day (S−)	/	Tot = 4817 S+ = 6066 S− = 2852		**Sedentary time (min/h of wear time):** Tot = 33.5 S+ = 32.3 S− = 36	**Total daily MVPA (min):** Total = 36.8/S+ = 49/S− = 11.1 **LPA (min/h of wear time):** Total = 23.3 (5.5)/S+ = 23.5 (5.8)/S− = 22.9 (5.0) **MVPA (min/h of wear time):** Total = 2.8 (0.1–9.5)/S+ = 3.5 (1.3–9.5)/S− = 0.8 (0.1–3.4) **Activity count (n):** Total = 330,500 (109,670–820,247)/S+ = 376,152 (168,845–820,247)/S− = 239,875 (109,670–365,337)
**Leavy et al., 2021** [21]	No comparison	/	5876 (3180)		**Sedentary time (min/d):** 598 (92)	**Brisk walking (min/d):** 23.5 (Q = 5.4–42.2) **LPA (min/d):** 197 (72) **MVPA (min/d):** 47 (34)
**Zajac et al., 2021** [28]	No comparison	/	7606.2 (3625.8)			

HC = healthy control. I = intervention. WD = weekdays. WE = weekend days. MLTA = Minnesota Leisure Time Activity. LPA = light physical activity. MVPA = moderate vigorous physical activity. HIPA = high intensity physical activity. mH = mHealth. AC = active control. HY = Hoehn and Yahr. MPALS = moderate-intensity lifestyle activities.
